# Protocol: Prospective observational study aiming for micro-elimination of hepatitis C virus in Nagawa town: The Nagawa Project

**DOI:** 10.1371/journal.pone.0256711

**Published:** 2021-08-26

**Authors:** Hiroyuki Kobayashi, Satoru Joshita, Yuki Akahane, Katsuhiko Matsuzaki, Hiromi Yamada, Daiki Aomura, Nao Joshita, Hajime Midorikawa, Kazuhiro Suyama, Masao Ota, Shun-ichi Wakabayashi, Yuki Yamashita, Ayumi Sugiura, Tomoo Yamazaki, Hiromichi Misawa, Takeji Umemura

**Affiliations:** 1 Division of Gastroenterology and Hepatology, Department of Medicine, Shinshu University School of Medicine, Matsumoto, Japan; 2 Department of Health Promotion Medicine, Shinshu University School of Medicine, Matsumoto, Japan; 3 Department of Clinical Laboratory, Yodakubo Hospital, Nagawa, Japan; 4 Department of Nephrology, Shinshu University School of Medicine, Matsumoto, Japan; 5 Health Care Center, Yodakubo Hospital, Nagawa, Japan; 6 Department of Internal Medicine, Yodakubo Hospital, Nagawa, Japan; 7 Spine Center, Yodakubo Hospital, Nagawa, Japan; 8 Consultation Center for Liver Diseases, Shinshu University Hospital, Matsumoto, Japan; 9 Department of Life Innovation, Institute for Biomedical Sciences, Shinshu University, Matsumoto, Japan; Kaohsiung Medical University, TAIWAN

## Abstract

**Background:**

The World Health Organization has set a goal of hepatitis C virus (HCV) elimination by the year 2030. However, no regions in Japan have succeeded in eradicating HCV. Micro-elimination is an approach to attain hepatitis C eradication in which national eradication goals are applied to specific populations so that viral treatment and control efforts can move forward quickly and efficiently. In order to eradicate HCV from Japan, this study aims to achieve HCV micro-elimination in the town of Nagawa.

**Methods and design:**

The Nagawa Project is an ongoing, prospective, multiple-institution, observational study running from April 1, 2021, to March 31, 2024. All residents of Nagawa town, excluding those under 20 years of age, not consenting to the study, or unable to undergo health check-ups due to nursing care needs, will be included. If found to be HCV antibody-positive, the participant will be recommended to see a doctor in consideration of MAC-2 binding protein glycosylation isomer values. Then, the participant will undergo serum HCV RNA measurement with the real-time polymerase chain reaction by an attending physician. If the participant is HCV RNA-positive, he or she will be referred to a hepatologist for further evaluation. In the case of a definitive diagnosis of chronic hepatitis C, direct acting antiviral treatment will be initiated. Through this process, HCV will be systematically micro-eliminated from the region.

**Discussion:**

The Nagawa Project will reveal the prevalence of chronic HCV in addition to the HCV eradication rate in Nagawa town towards achieving HCV micro-elimination.

**Trial registration:**

This study is performed by Shinshu University School of Medicine and was registered as UMIN 000044114 on May 6, 2021.

## Introduction

Hepatitis C is a form of liver disease caused by the hepatitis C virus (HCV). An estimated 71 million people globally and 1 million people in Japan have chronic HCV infection. Chronic infection leads to liver cirrhosis and eventually to hepatocellular carcinoma (HCC) [1], and remains the primary cause of liver cirrhosis and cancer in Japan [2]. However, advances in such therapeutic agents as direct acting antivirals (DAAs), with sustained virological response (SVR) rates of over 95% [3, 4], have reached a stage where it is possible to eliminate HCV entirely.

The World Health Organization (WHO) has announced its commitment to eradicate HCV by the year 2030 via a series of therapeutic measures. However, assuming that high-income countries maintain their current levels of diagnosis and treatment, only 24% are on track to eliminate HCV by 2030, and 60% are off track by at least 20 years [5, 6]. Among developed nations, Japan appears to be on schedule to meet the WHO’s HCV elimination target and should maintain its current levels of HCV diagnosis and treatment. However, improved screening and management remain crucial to ensure the WHO’s goal is met. Specifically, chronic HCV infection is often undiagnosed because it remains largely asymptomatic until the progression to liver cirrhosis or HCC, which highlights the importance of screening and early detection.

In addition to preventing primary and secondary infections, three steps have been outlined to eradicate HCV: 1) Examination to identify HCV-positive individuals through HCV testing, 2) Consultation after encouraging HCV-positive individuals to see hepatologists at medical institutions, and 3) Treatment by eradicating HCV with appropriate DAA interventions. These policies are carried out based on the Basic Act on Hepatitis Measures in Japan. However, from the viewpoint of total HCV eradication, the current rates of HCV testing, medical institution consultations, and treatment may be insufficient; in fact, no region in Japan has successfully eradicated HCV.

"Micro-elimination" is an approach that seeks to quickly and efficiently enable treatment and prevention by applying national-level eradication goals to specific populations, such as certain regions or people with certain demographics [7]. In order to eradicate HCV, it will be important to first perform HCV testing on everyone in a regional cohort (i.e., Examination), then increase the rate of secondary examinations by motivating people testing positive to seek medical advice (Consultation) and receive DAAs (Treatment). Achieving micro-elimination in this way is considered the first step towards eradicating HCV in Japan.

The rural town of Nagawa in the Chiisagata district of Nagano Prefecture (population: 5,903 as of September 1, 2020; mayor: Kenichiro Hata) has been pursuing an aggressive health check-up initiative since May of 1972 under the municipal government’s Children and Health Promotion Division. A system has been created that allows residents to undergo a so-called “Ningen Dock” general health check-up and a metabolic syndrome-specific check-up in alternating years, with certain examinations provided at no cost to the individual. The town’s only public medical institution is Yodakubo Hospital (https://www.yodakubo-hp.jp/), at which all extensive health check-ups are carried out. The metabolic syndrome-specific health check-ups are performed as part of mobile health screenings in every town district with the assistance of the hospital. In addition, many residents with chronic illnesses visit Yodakubo Hospital on a regular basis. Based on the above circumstances, Nagawa town is considered a suitable candidate for the “Nagawa Project” for HCV micro-elimination.

Developed in Japan, MAC-2 binding protein glycosylation isomer (M2BPGi) is a novel serum glycan marker whose secretion from hepatic stellate cells increases with hepatic fibrosis progression [8]. We have described the clinical usefulness of M2BPGi for diagnosing liver fibrosis in chronic liver disease and demonstrated its usefulness as a prognostic biomarker in liver disease and cancer [9–12]. Therefore, the inclusion of M2BPGi results may help in motivating otherwise asymptomatic HCV-positive patients to seek further medical consultation and commence DAA treatment.

The current study has three main goals: 1) complete the screening of all adult residents in Nagawa town and identify the prevalence of HCV RNA-positive individuals, 2) motivate HCV-positive residents to receive consultation at medical institutions with support of M2BPGi findings, and 3) provide DAA intervention and identify the rate of HCV eradication. Ultimately, the Nagawa Project aims to achieve HCV micro-elimination in the town.

## Materials and methods

### Study design

**Fig 1:**
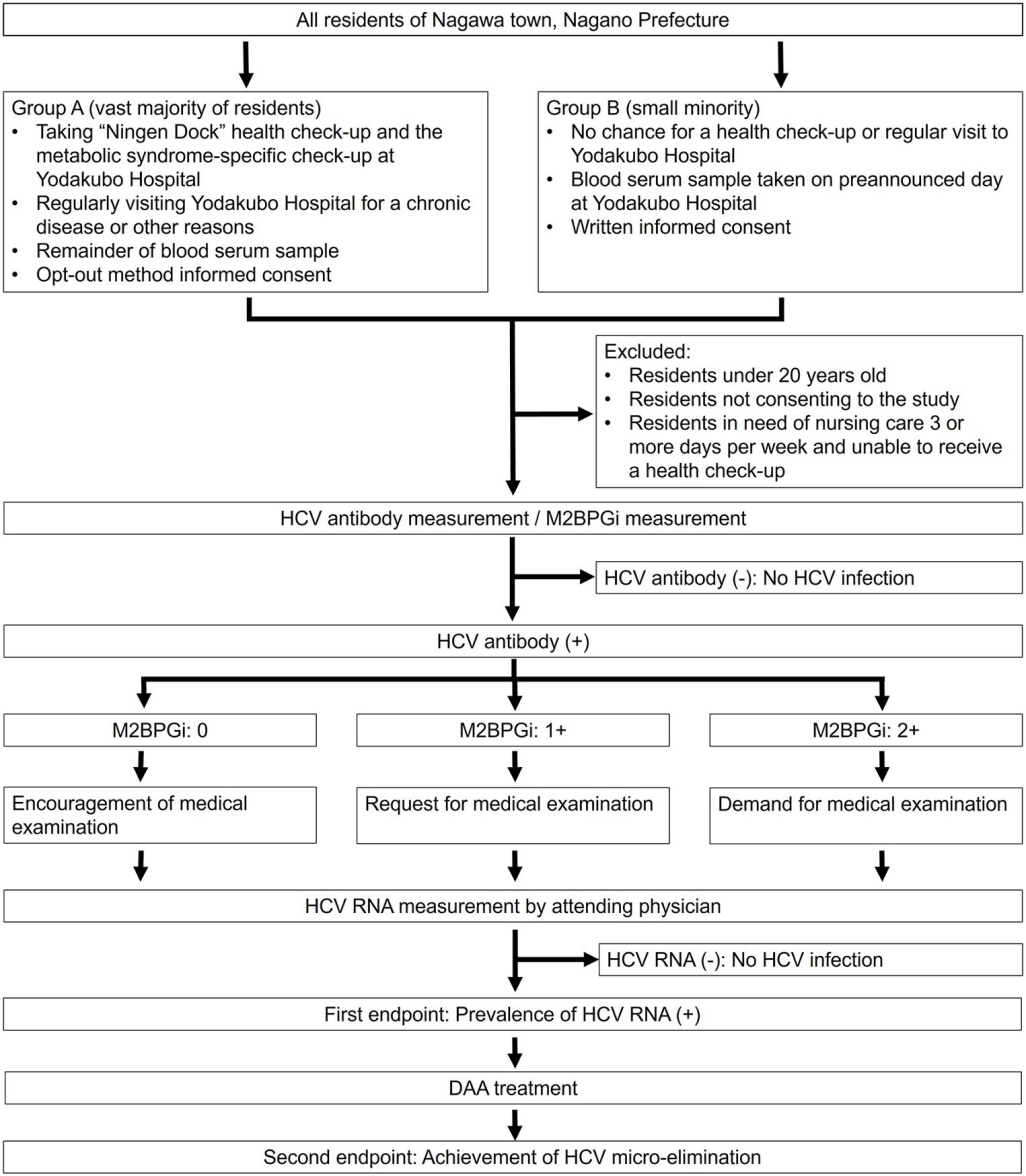
The study flowchart of the Nagawa Project. Abbreviations: HCV, hepatitis C virus; M2BPGi, MAC-2 binding protein glycosylation isomer.

The Nagawa Project is an ongoing, prospective, multiple-institution, observational study that will run from April 1, 2021, to March 31, 2024. The project flowchart is presented in Fig 1. This investigation was reviewed and approved by the Institutional Review Board of Shinshu University School of Medicine (approval number: 4992) on January 5, 2021, and of Yodakubo Hospital (approval number: 2020–8) on January 26, 2021. Both Institutional Review Boards have approved the use of opt-out consent, as described below.

### Ethical statement

All researchers involved in the Nagawa Project will conduct the study in accordance with the Declaration of Helsinki (revised in 2013 by Fortaleza) and the Ethical Guidelines for Medical Research Involving Human Subjects (partially revised on February 28, 2017). An opt-out system is in place for participants in Group A (described below), while written informed consent is obtained from participants in Group B, as shown in Fig 1 and Table 1. All information on the protocol and conduct of the study, including its purpose, is available on the Yodakubo Hospital website (https://www.yodakubo-hp.jp/) and at the hospital reception counter. If patients do not wish to participate in the research, they are freely able to opt out of the study.

**Table 1:** Method for obtaining blood samples and informed consent in the Nagawa Project.

Group A (vast majority)	Residents taking the “Ningen Dock” health check-up or a metabolic syndrome-specific check at Yodakubo Hospital
	Residents regularly visiting Yodakubo Hospital for a chronic disease or other reason
Blood sample	Remainder of blood serum sample taken for laboratory testing
Informed consent	Opt-out method
Group B (small minority)	Residents with no chance for a health check-up or regular visit to Yodakubo Hospital
Blood sample	Blood serum sample taken on preannounced day at Yodakubo Hospital
Informed consent	Written informed consent

### Target population

#### Inclusion and exclusion criteria

All residents of Nagawa town were initially targeted. Group A (vast majority of residents) consisting of people who undergo the “Ningen Dock” health check-up and the metabolic syndrome-specific check-up at Yodakubo Hospital or patients who regularly visit Yodakubo Hospital for a chronic disease or other reasons and Group B (minority of population) including individuals unable to visit the hospital for the reasons described for Group A above are included. The exclusion criteria are as follows: 1) under 20 years of age, 2) not consenting to the study, and 3) in need of nursing care three or more days per week and unable to receive a health check-up. The remainder of the residents of Nagawa town will be included in the study cohort as shown in Fig 1.

### Collection of blood serum samples (Fig 1 and Table 1)

- Group A: The remainder of blood samples (serum) taken for laboratory testing is stored for analysis.
- Group B: A blood sample will be taken on a preannounced day at Yodakubo Hospital specifically for this population.

#### Trial design and follow-up

Participant registration for the study will run from June 1, 2021, to September 30, 2023. All participants will undergo HCV antibody II (HCVAb) testing (Sysmex Co., Kobe, Japan) and M2BPGi (Sysmex Co., Kobe, Japan) measurement with a HISCL-5000 (Sysmex Co., Kobe, Japan) system using stocked serum. An HCVAb test result ≥1.00 cutoff index (COI) is considered a positive result. M2BPGi readings of <1.00 COI, ≥1.00 COI and <3.00 COI, and ≥3.00 COI, are judged as negative, 1+, and 2+, respectively. Regarding linkage-to-care, if an HCV-positive patient has a high M2BPGi value, this will be used to explain that the risk of cirrhosis and HCC is high at present, and early consultation and treatment will be urged. On the other hand, if M2BPGi is low in an HCV-positive patient, clinicians will make the individual aware of the possibility of progression to cirrhosis and HCC with the onset of fibrosis, at which point M2BPGi will being to rise, and that treatment is crucial to prevent further M2BPGi increases. When the M2BPGi results are returned, an explanatory document with the above descriptions is also given to the participants to encourage them to undergo additional medical examination. The test results will be delivered by mail within two weeks after the analysis. In addition, HCV-positive patients who have not visited the hospital within a few months who receive a health check-up will be encouraged by the town to see a doctor. Such patients who regularly visit Yodakubo Hospital will be recommended by the doctor in charge to see a liver specialist. If the HCVAb test is positive, the participant will be recommended to consult a doctor in consideration of M2BPGi values, at which time serum HCV RNA measurement by the real-time polymerase chain reaction will be performed by an attending physician. If the participant is HCV RNA-positive, he or she will be referred to a hepatologist for further evaluation, whereby a definitive diagnosis of chronic hepatitis C will be ascertained according to diagnostic criteria by the Japan Society of Hepatology [13]. In such cases, DAA treatment will be initiated with patient consent by a hepatologist based on the guidelines of the Japan Society of Hepatology [13]. Patients will be monitored for a SVR every six months to calculate the SVR achievement rate in Nagawa town. Ultimately, the final HCV eradication rate will be determined on March 31, 2024.

#### Study endpoints

The first endpoint in this study is determination of the prevalence of chronic hepatitis C patients in Nagawa town. The second endpoint is the rate of HCV elimination in Nagawa town on March 31, 2024.

#### Safety

In the Nagawa Project, blood samples will be collected during medical check-ups, with no additional intervention or invasiveness. Moreover, this study will be conducted free of charge and thus pose virtually no financial burden on the subjects. Therefore, the risk of non-participation is considered to be very low.

### Statistical considerations

#### Sample size estimation

We plan to screen all adult residents in the town of Nagawa (approximately 4500). Since the HCV-positive rate is reportedly 0.35% among workers in Japan [14], it is estimated that approximately 16 people are HCVAb-positive.

#### Statistical analysis

The HCVAb-positive rate and the HCV RNA-positive rate will be examined. In gender and age comparisons, continuous variables will be statistically evaluated by means of the Mann–Whitney U test, while categorical variables will be analyzed using the chi-square test in order to detect background differences. The HCV elimination rate will be examined at the end of the study.

## Discussion

The Nagawa Project is a prospective observational study that will screen all eligible residents for HCV infection and ultimately aims to achieve HCV micro-elimination in the town of Nagawa.

In spite of dramatic advances in DAA treatment for HCV, HCV micro-elimination has yet to be attained in any area of Japan. One possible reason is an unawareness of infection due to the asymptomatic nature of the liver until considerable disease progression, indicating that an HCVAb test is essential to learn of HCV infection. Therefore, in order to achieve micro-elimination, it is essential to establish a system in which all local residents can receive HCV testing. Nagawa town has been actively promoting health check-up services for decades and has established a system that allows residents to receive routine examinations at Yodakubo Hospital. In addition, many residents with chronic diseases visit the hospital on a regular basis. We examined the percentage of Nagawa town residents who had a health check-up or outpatient visit in 2019. Among patients using national health insurance and among the very elderly, the majority had the opportunity for a medical check-up or an outpatient blood test. In the remaining younger cases and residents using employee’s health insurance provided by the Social Health Insurance Unions and the Health Insurance Association of Japan (i.e.: Kyokai Kenpo), at least 46% had a medical check-up at Yodakubo Hospital for a chronic disease, although some residents underwent medical check-ups at other facilities. In our protocol, the township will identify all the residents, and they will be able to receive the test free of charge at their regular medical institutions. For those who do not have regular visits to the hospital, all residents will be given the opportunity to be tested by issuing a request form. Moreover, we offer preannounced days for free blood sample collection at Yodakubo Hospital for this minor population to attain 100% HCVAb testing. In addition, depending on the results, DAA treatment is also available. In order to increase a participating rate of 100%, this study is cooperated with the government of Nagawa town as well as Yodakubo hospital. Nagawa town can identify all its residents by their resident cards and town insurance cards. The insurance card makes it possible to ascertain whether a person has visited a hospital or had an examination. By issuing documents confirming that the residents have been inspected, it is possible to inform residents who have not been inspected and allow those who wish to be inspected to do so. In this way, all adults can be tested.

Another possibility is that even if individuals know of their HCV infection, some avoid further consultation and DAA treatment in the absence of symptoms. Of the 1028 people in Nagawa town who underwent health check-ups in 2018, 139 (14%) received the HCVAb test. Three people were HCVAb-positive, among whom only two were recorded as having a secondary evaluation and had already been achieved an SVR by prior antiviral treatment. In order to motivate patients to action, the present study includes M2BPGi measurements to help visualize liver fibrosis stage. M2BPGi is low in healthy individuals and becomes significantly elevated with increasing liver fibrosis, and furthermore is associated with the development of HCC, liver-related complications, and prognosis [15–17]. Therefore, M2BPGi may be useful both as a first-line screening method in health check-ups as well as a motivational tool for suspected patients to undergo secondary screening. In order to achieve HCV eradication nationwide in Japan, more effective tactics will be needed.

Lastly, public awareness campaigns to share information on HCV will be important to maximize the examination and consultation rates. If the examination rate is too low, further information will be provided via the "Koho Nagawa" town publication and on Nagawa town cable television with the assistance of the Nagawa town government. In addition, we will promote the tests conducted in the Nagawa Project via hepatitis medical coordinators in the community. Residents who visit Yodakubo Hospital will also be given information via the hospital’s public relations magazine "Yodakubo Hospital Dayori".

Based on the above mentioned, it is highly likely that micro-elimination in the community can be achieved if this protocol is followed. For the residents of Nagawa town, the HCV test will enable earlier detection and initiation of eradication therapy, which will increase healthy life expectancy. As a downstream effect, this will also contribute to a reduction in future medical costs. In addition, once HCV micro-elimination in Nagawa town is declared, it will create a roadmap and greater opportunity for eliminating HCV in other parts of Nagano Prefecture and across Japan, with the goal of HCV elimination nationwide.

## Conclusions

The Nagawa Project will reveal the prevalence of chronic hepatitis C patients as well as the HCV eradication rate in Nagawa town towards achieving HCV micro-elimination across Japan.

## Abbreviations

COI: cutoff index
DAA: direct acting antiviral
HCC: hepatocellular carcinoma
HCV: hepatitis C virus
M2BPGi: MAC-2 binding protein glycosylation isomer
SVR: sustained virological response
